# Reporting Clinical Outcomes in Hand Surgery Randomized Controlled Trials: A Systematic Review Using Wide-Awake Local Anesthesia No Tourniquet Studies as a Model

**DOI:** 10.1016/j.jhsg.2024.08.007

**Published:** 2024-09-19

**Authors:** Jad Lawand, Ashraf Hantouly, Fadi Bouri, Mohammad Muneer, Elisabet Hagert

**Affiliations:** ∗ University of Texas Medical Branch, Galveston, TX; † Department of Orthopedic Surgery, Hamad Medical Cooperation, Doha, Qatar; ‡ Department of Plastic Surgery, Hamad Medical Corporation, Doha, Qatar; § Department of Surgery, Aspetar Orthopaedic and Sports Medicine Hospital, Doha, Qatar; ‖ Department of Clinical Science and Education, Karolinska Institutet, Stockholm, Sweden

**Keywords:** Clinical outcomes, Hand surgery, Patient-reported outcomes, Randomized controlled trials, WALANT

## Abstract

**Purpose:**

The purpose of this study was to comprehensively evaluate clinical outcome reporting in hand surgery randomized controlled trials (RCTs), using wide-awake local anesthesia no tourniquet (WALANT) studies as a model.

**Methods:**

This International Prospective Register of Systematic Reviews-registered systematic review (CRD42023461653) adheres to preferred reporting items for systematic reviews and meta-analysis guidelines, focusing on RCTs evaluating WALANT in hand and upper limb surgery. A systematic search across five databases was conducted to include all eligible articles from inception until search date (April 1, 2023). Inclusion criteria encompassed WALANT RCTs in upper limb surgery, with exclusion criteria addressing non-RCTs and non-English studies. Data extraction covered study characteristics, patient demographics, procedures performed, and outcomes reported. The revised Cochrane risk-of-bias tool for randomized trials was employed for quality assessment.

**Results:**

The search identified 304 articles—after screening, 11 were included for analysis, encompassing 889 patients in WALANT RCTs. Technical outcomes were most reported (73%), whereas functional was least commonly reported (36%). The analysis encompassed a heterogeneous patient cohort, with an average follow-up period of 41.3 days. Challenges in standardizing functional outcomes and patient-reported outcomes were identified. The Cochrane risk-of-bias tool for randomized trials indicated an overall low risk, affirming the methodological rigor of the included studies.

**Conclusions:**

A significant diversity in outcome reporting and assessment tools was identified, emphasizing the challenges in standardization and outcome reporting across RCTs. Although technical outcomes were prevalent, patient-reported and functional outcomes were often lacking. The study underscores the need for further research standardization to optimize patient care and advance evidence-based decision making, as variability in outcomes reporting hinders the ability to draw consistent conclusions and comparisons across studies.

**Type of study/level of evidence:**

Therapy/Prevention, Etiology/Harm IA.

Well-conducted randomized clinical trials (RCTs) hold a paramount position in the hierarchy of medical evidence, serving as the gold standard for evaluating the efficacy and safety of medical interventions.^1^ Randomized clinical trials are considered fundamental to medical advancement, as they provide evidence enabling health care professionals and researchers to make informed decisions to guide patient care. In hand surgery, the resurgence of wide-awake local anesthesia no tourniquet (WALANT) has transformed hand surgery by enabling surgeons to forego the use of a tourniquet and perform operating theater cases in procedure rooms.^2^, ^3^, ^4^

Epinephrine was previously thought to be contraindicated in procedures involving the extremities because of concerns about potential vasoconstriction and tissue compromise.^5^ However, recent developments in the medical literature have refuted this epinephrine myth,^6^ and a recent systematic review on the safety and efficacy of WALANT in hand surgery has shown the low inherent risks of the technique.^7^ As part of the establishment of WALANT in general hand surgery practice, the technique has been used in multiple RCTs, evaluating both technical and patient outcomes.

Despite the wealth of evidence emerging from the medical literature, the quality of RCTs assessing the safety, efficacy, and overall outcomes of hand surgery remains an area of concern. Randomized clinical trials are recognized for their ability to minimize bias and confounding variables, offering a more rigorous and systematic approach to assessing the true impact of a medical intervention. However, key challenges in the assessment of outcomes reported across RCTs prevail, with a diversity of end points and measurement methods making it challenging to synthesize and compare results effectively.

The purpose of this systematic review was to address these critical issues by conducting a thorough evaluation of existing hand surgery RCTs, using WALANT studies as a model. The aim was to provide insights into the current state of knowledge and patterns of outcome reporting in RCTs and identify areas where further research and standardization are needed to optimize patient-related outcomes and subsequent care in hand surgery.

## Materials and Methods

Our International Prospective Register of Systematic Reviews-registered CRD42023461653 systematic review was conducted with strict adherence to the preferred reporting items for systematic reviews and meta-analyses. This systematic review focused on the type of outcomes reported by WALANT RCTs in the upper limb literature.

### Search strategy

A systematic search was conducted on April 1, 2023, across five databases (OVIDMedline, Embase, Web of Science, Cochrane, and Scopus) to screen all the studies from inception till search date. Broad keywords were used: “wide-awake surgery” OR “wide awake surgery” OR “wide awake without tourniquet” OR “local anesthetic no tourniquet” OR “local anesthetic without tourniquet” OR (“RCT” AND “WALANT”) OR (“randomized control trial” AND “WALANT”).

#### Inclusion criteria

This study included only RCTs that specifically reported the use of WALANT in hand and/or upper limb surgery.

#### Exclusion criteria

Non-RCTs, non-English studies, non–peer-reviewed articles, and review studies were excluded.

#### Study screening

**Figure 1:**
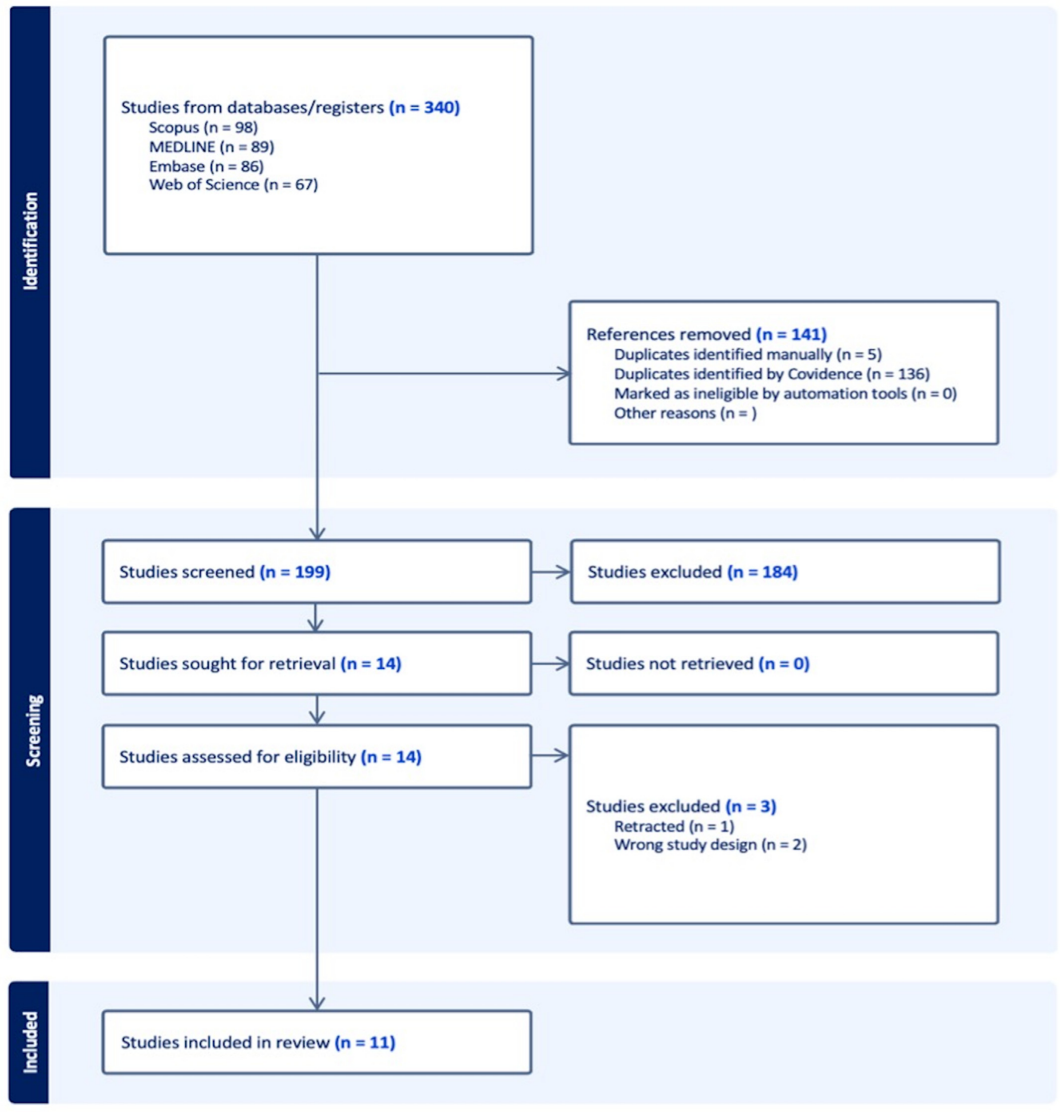
Preferred reporting items for systematic reviews and meta-analyses flow diagram for the selection of upper limb WALANT RCTs.

An independent and blinded screening process was carried out by two authors. The screening comprised two distinct phases, with an initial screening phase based on titles and abstracts, followed by a full-text review phase. Any disagreement between the two authors was solved by a discussion with the senior author (Fig. 1). The primary outcome was classified into five domains functional, patient-reported outcomes (PROs), administrative, technical, and complications. Secondary outcomes examined the modality and protocol-specific administration of these domains.

#### Data abstraction and data items

Two authors independently and blindly extracted the data, followed by a cross-checking process to ensure accuracy. The following data items were collected: study characteristics, patient demographics, procedures performed, functional outcomes, PROs, administrative, technical, complications monitored or reported, and administrative outcomes. Functional outcomes refer to assessments and measurements of hand function/and or disability. Patient-reported outcomes encompassed assessments related to the patient’s experience and outcomes using standardized questionnaires. Technical outcomes pertained to outcomes specific to surgical technique. Administrative outcomes included benefits relevant to the health care system, encompassing aspects such as surgical logistics. Additionally, the exact method of reporting and assessments used to quantify the five domains of interest was documented.

#### Risk-of-bias of the included studies

**Figure 2:**
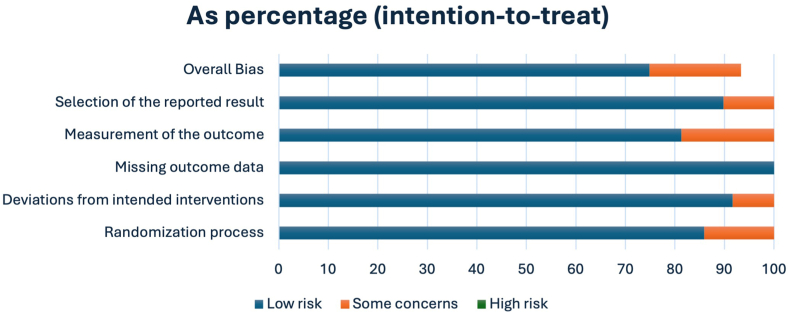
Cochrane risk-of-bias 2 quality assessment of included studies.

Quality assessment of the included studies was performed by two authors blindly and independently using the revised Cochrane risk-of-bias tool (Fig. 2) for randomized trials (RoB-2).^8^ Any discrepancy between the two authors was resolved by a discussion with the senior author to reach a consensus.

## Results

### Study selection

The electronic search identified 304 studies, of which 141 duplicates were excluded, leaving 199 studies for manual title and abstract screening (Fig. 1). A total of 11 RCTs focusing on WALANT were finally included (±. 1).^9^, ^10^, ^11^, ^12^, ^13^, ^14^, ^15^, ^16^, ^17^, ^18^, ^19^

### Study characteristics

The total number of included patients was 889 from 11 RCTs, 476 of whom were randomized to the WALANT cohort. The median number of total patients enrolled in each study was 72, with an interquartile range (IQR) from 47 to 185. Within the WALANT group, 195 (23%) patients were men. The mean follow-up period was 41.3 days, (median: 12.5; IQR: 2–180 days). A full depiction of study characteristics is displayed in Table 1.

**Table 1:** Characteristics of RCTs on Upper Limb WALANT

Study Demographics									
Authors, Country, and Year	Total Patients	Mean WALANT Age	Experimental Group	Control	WALANT Solution		Use of Sodium Bicarbonate	WALANT Group Sample Size (M)	Follow-Up Period (Days)
					% Lidocaine/Equivalent, mL	[Epinephrine], mL			
Abdelshaheed, Mohamed Elsaid, 2022, UK	52	27	WALANT	Wide-awake local anesthesia initially applied tourniquet	2%, 0.5% bupivacaine, -	1:100,000, -	-	26 (21)	2
Bashir et al, 2015, India	75	30	WALANT	WALANT with different time from injection to incision	2%, 20	1:1,000, 1	-	75 (59)	0
Gunasagaran et al, 2017, Malaysia	40	61.4	WALANT	LA/Tourniquet	1%, 100	1:100,000, 1	Y	20 (3)	2
Hoxhallari et al, 2019, USA	41	NA	WALANT	WALANT with virtual reality	-, -	-, -	-	41 (18)	NA
Iqbal et al, 2018, UK	73	64.2	WALANT	LA/Tourniquet	1%, -	1:200,000, -	-	36 (14)	90
Ki Lee S, Gul Kim S, Sik Choy W, 2020, Korea	185	52.7	WALANT	LA/Tourniquet	2%, 50	1:1,000, 1	Y	94 (26)	2
Ki Lee et al, 2022, Korea	169	59.31	WALANT	LA/Tourniquet	1%, 100	1:1,000, 1	Y	56 (19)	180
Okamura et al, 2021, Brazil	72	51.6	WALANT	Biers Block	1%, -	1:100,000, -	-	38 (0)	84
Prasetyono et al, 2022, Indonesia	35	32	WALANT	LA/Tourniquet	0.2%, -	1:1,000,000, -	-	17 (5)	20
Ruxasagulwong et al, 2015, Thailand	60	55.3	WALANT	LA/Tourniquet	2%, -	1:80,000, -	-	30 (30)	28
Mohd Rashid et al, 2019, Malaysia	86	NA	WALANT	LA/Tourniquet	1% , -	1:100,000, -	Y	43 (NR)	5

LA, local anesthesia.

### Quality assessment

The Cochrane RoB-2 tool was employed to assess the ROB in the included studies, with weights assigned based on methodological rigor and relevance (Fig. 2). Most studies were found to have a “low” ROB in most domains, indicating strong methodological quality. However, “some concerns” were raised in specific domains for three studies, drawing attention to potential bias sources in “deviations from intended interventions,” “selection of the reported result,” and overall bias.^9^, ^10^, ^11^ The overall bias assessment revealed that most studies were rated as “low,” signifying an overall low ROB (±. 2).

### Functional outcomes

Functional outcomes were the least frequently reported among the various domains assessed with only four of the 11 included studies reporting functional outcomes.^12^, ^13^, ^14^, ^15^ This subset of studies included 349 patients, 147 of whom received treatment with WALANT. Each of these studies randomized a median of 37 patients (IQR: 31.25–58). The median follow-up reported across the subset of studies reporting functional outcomes was 87 days (IQR: 68–180). Additional studies assessed the total active and passive range of motion both before surgery and at 3 months after surgery.^14^ A full breakdown of functional outcomes reported can be found in Table 2.

**Table 2:** Functional Outcome Assessments Employed in Upper Limb WALANT RCT

Functional Outcomes								
Authors, Year	Procedures Performed	ROM	DASH	Levine SSS	Levine FSS	Boston Questionnaire	Quinnell Grade	Historical Objective (Hi-Ob) Scale
Iqbal et al 2018	CTR	-	Y	Y	Y	-	-	-
Ki Lee et al, 2022, Korea	CTR	-	Y	-	-	-	Y	Y
Okamura et al 2021	burn contracture release	-	-	-	-	Y	-	-
Prasetyono et al 2022	TFR, CTR, and de Quervain’s disease	Y	-	-	-	-	-	-

CTR, Carpal tunnel release; TFR, Trigger finger release; ROM, Range of motion; DASH, Disabilities of the arm shoulder and hand; SSS, Symptom severity score, FSS, Functional severity score.

### Patient-reported outcomes

Among the 11 studies examined, seven provided data on PROs, involving a total of 633 patients, with 311 undergoing WALANT intervention. The median number of patients randomized to groups was 72, with an IQR from 47 to 185. Within the WALANT group, the median number of patients was 38, with an IQR of 31 to 94. Men constituted 32% of all WALANT patients. The Likert and visual analog scales were the most frequently used assessment tools for outcomes. Anxiety levels were assessed using the Hamilton Anxiety Rating Scale and the Hospital Anxiety and Depression Scale. Data collection for these outcomes spanned the perioperative period, encompassing assessments from intraoperative to up to 2 days after surgery. A full list of included PRO is presented in Table 3.

**Table 3:** Patient-Reported Outcomes Reported in Upper Limb WALANT RCT

Patient-Reported Outcomes						
	Anxiety	Satisfaction	Patient-Reported Outcomes Instrument	Pain		Perceived Comfort
Abdelshaheed, Mohamed Elsaid, 2022, UK	N	Y	VAS	Intraoperative	After surgery	N
				Y	N	
Gunasagaran et al, 2017, Malaysia	N	N	Likert Scale	Intraoperative	After surgery	Y
				N	N	
Hoxhallari et al 2019	Y	Y	VAS	Intraoperative	After surgery	Y
				Y	N	
Iqbal et al, 2018, UK	N	N	VAS	Intraoperative	After surgery	Y
				Y	Y	
Ki Lee S, Gul Kim S, Sik Choy W, 2020, Korea	N	Y	VAS, Surgery-related anxiety was assessed using the Hamilton Anxiety Rating Scale (HAM-A).	Intraoperative	After surgery	N
				Y	Y	
Ki Lee et al, 2022, Korea	Y	Y	VAS	Intraoperative	After surgery	N
				Y	Y	
Okamura et al, 2021, Brazil	Y	N	VAS	Intraoperative	After surgery	N
				Y	Y	

Y, yes; N, no.

### Administrative outcomes

Among the 11 studies analyzed, seven explored one or more administrative outcomes of WALANT in RCTs, as detailed in Table 2. The total number of patients across these studies was 679, with a median of 80.5, and an IQR from 62 to 185. Of the seven studies, all of them reported surgical time, and three studies provided preoperative preparation data, including the time from WALANT injection to anesthetic effect onset. Within the subset of patients who received WALANT intervention in studies reporting administrative outcomes, there were a total of 352 participants with a median number of 50 patients (IQR: 32–94). Men patients accounted for approximately 37% of patients treated with WALANT. The studies, on average, had a median follow-up of 3.5 days and an IQR from 2 to 84 days. A full list of PROs can be found in Table 4.

**Table 4:** Administrative Outcomes Reported in Upper Limb WALANT RCT

Administrative Outcomes			
Authors, country, and year	Surgical time	Preoperative preparation time	Time taken to anesthetize
Abdelshaheed, Mohamed Elsaid, 2022, UK	Y	N	N
Gunasagaran et al, 2017, Malaysia	Y	Y	Y
Ki Lee et al, 2022, Korea	Y	Y	N
Bashir et al, 2015, India	Y	N	Y
Ki Lee S, Gul Kim S, Sik Choy W, 2020, Korea	Y	Y	N
Okamura et al, 2021, Brazil	Y	N	N
Mohd Rashid et al, 2019, Malaysia	Y	N	Y

Y, yes; N, no.

### Technical outcomes

Of the 11 studies analyzed, eight reported technical outcomes. The technical outcomes were assessed in a total of 595 patients, with a median of 72 randomized patients (IQR: 41.5–185.4); of these, patients were randomized to a WALANT intervention, with a median of 40 patients (IQR: 28–94). Among the WALANT patients, 39% were men, accounting for 141 patients. The mean follow-up period for these technical outcomes had a median of 5 and an IQR from 2 to 180 days. Surgical visibility was the most reported technical outcome with five of eight studies reporting it, whereas postoperative anesthetic time was only reported by one study as detailed in Table 5.

**Table 5:** Technical Outcomes Reported in Upper Limb WALANT RCT

Technical						
Authors, Country, and Year	Duration of the Anesthetic Effect	Surgical Viability	EBL	Time to Anesthetic Effect	Vital Changes	Postoperative Duration of Anesthetic Effect
Bashir et al, 2015, India	N	Y	N	Y	N	N
Gunasagaran et al, 2017, Malaysia	N	N	Y	N	N	N
Ki Lee, et al, 2020, Korea	Y	Y	N	N	N	N
Prasetyono et al, 2022, Indonesia	N	Y	N	N	N	Y
Hoxhallari et al, 2019	N	N	N	N	Y	N
Okamura et al, 2021, Brazil	N	N	N	N	N	N
Ruxasagulwong et al, 2015, Thailand	N	Y	Y	N	Y	N
Mohd Rashid et al, 2019, Malaysia	Y	Y	N	Y	N	N

EBL, Estimated Blood Loss.

### Complications

Among the 11 studies examined, six provided data on complications, involving a total of 385 patients, with 232 undergoing WALANT surgery. The median number of patients randomized to groups was 66, with an IQR of 54–86. Within the WALANT group, the median number of patients was 34 (IQR: 27–75). Men constituted 49% of all WALANT patients. Of the six studies that assessed complications, 50% did not specify which complications were monitored. Median follow-up was 1 day (IQR: 0.5–2).^9^, ^10^, ^11^^,^^15^^,^^18^ Conversion to general anesthesia is the most commonly predefined complication in upper limb WALANT RCTs, and it was mentioned in 50% of all studies, as shown in Table 6.^9^^,^^10^^,^^15^

**Table 6:** Included Studies and Reported Complications in Upper Limb WALANT RCT

Complication Monitored					
	Unspecified	Vascular	Revisions/Failures	Infection	Conversion to GA
Abdelshaheed, Mohamed Elsaid, 2022, UK	-	-	-	-	Y
Bashir et al, 2015, India	-	-	-	-	Y
Gunasagaran et al, 2017, Malaysia	Y	-	-	Y	-
Okamura et al., 2021, Brazil	-	-	Y	-	Y
Ruxasagulwong et al, 2015, Thailand	Y	Y	-	-	-
Mohd Rashid et al, 2019, Malaysia	Y	-	-	-	-

### Outcome definition and timing variation

Of the 11 studies included in our analysis, 10 provided data on follow-up periods, which exhibited an average duration of 41.3 days (median: 12.5 days; IQR: 2–180 days; Table 1).

## Discussion

This systematic review examined the outcomes documented in published RCTs, using WALANT as a model. Outcomes assessed included functional, PRO, administrative, technical, and complications, accounting for 36%, 64%, 64%, 72%, and 55% of all included RCTs, respectively. Well-executed RCTs play a crucial role in advancing our comprehension of WALANT and informing evidence-based practices; however, the lack of uniformity in the outcomes reported may have hindered the advancement of knowledge in this field.

The generalizability of clinical trial data depends on the extent to which the study population, interventions, and outcomes align with broader patient populations and real-world scenarios.^20^ This consideration is vital for ensuring that findings from controlled trials are applicable and relevant beyond the specific conditions of the study. In hand surgery, the fulfillment of preoperative patient expectations and effective pain relief are recognized as key factors influencing postoperative patient satisfaction.^21^ Consequently, clinicians often turn to the literature to gauge and manage patient expectations. The functional outcomes of patients treated under WALANT are crucial in considering the utility of this technique in clinical practice. However, a notable limitation in this regard is the insufficient availability of such data within RCTs, posing a significant challenge for clinicians.

Furthermore, additional studies examining clinical outcomes have identified a notable gap characterized by inconsistency and heterogeneity in the reporting of functional outcomes, preventing conclusions on the efficacy of intervention.^22^^,^^23^ This variability in functional outcomes reporting hinders the ability to draw consistent conclusions and comparisons across different studies, emphasizing the need for standardized and comprehensive reporting mechanisms to enhance the utility of research. Therefore, acknowledging the limitations and potential variations from real-world scenarios is crucial for making informed and personalized decisions in clinical practice.

Patient-reported outcomes are increasingly recognized as valuable measures in assessing the impact of hand surgery on patients' lives. However, despite the growing emphasis on capturing PROs, our review identified heterogeneity in the assessment tools employed across studies. This makes it difficult to compare results across RCTs, limiting the ability to draw meaningful conclusions on the overall effectiveness and patient experiences associated with various orthopedic interventions. The most frequently used assessment tools for outcomes identified in the study are the Likert and visual analog scales, both employed in 38% of studies reporting PROs to evaluate pain, discomfort, and/or patient satisfaction. This issue has been identified as ambiguity in consolidated standards of reporting trials (CONSORT), forming the basis for adopting a modified CONSORT PRO extension with the goal of improving valuable PROs.^24^

Psychometric conversions, where properties of different PROs are compared and analyzed, can aid in facilitating more meaningful comparisons and meta-analyses in instances where multiple PRO assessments are conducted. By standardizing PROs through psychometric conversions, researchers and clinicians could gain a more comprehensive understanding of patient experiences and treatment outcomes.^25^ To date, however, there are numerous challenges and limitations in comparisons of commonly used PROs, with Disabilities of the Arm Shoulder and Hand, quick Disabilities of the Arm Shoulder and Hand, and Michigan Hand Questionnaire being the best described regarding psychometric properties.^25^ A more cohesive understanding of PROs could be achieved through focused psychometric conversions, ultimately contributing to improved evidence-based decision making in hand surgery.

Wide-awake local anesthesia no tourniquet offers the unique ability to shift procedures from a traditional operating room onto minor procedure rooms, improving resource allocation.^4^^,^^26^ This benefit has been well-characterized in the literature; however, among the 73% of WALANT RCTs reporting administrative benefits in preoperation preparation time, times from WALANT injection to anesthetic effect were most reported.

In the context of WALANT, the distinctive capability to transition procedures from traditional operating rooms to minor procedure rooms provides a distinct advantage by enhancing resource allocation.^4^^,^^26^ This well-documented benefit aligns with the broader literature on WALANT and underscores its potential impact on optimizing health care resources. Notably, among the subset of WALANT RCTs that reported administrative benefits (comprising 64% of the studies), preoperative preparation time and the duration from WALANT injection to anesthetic effect emerged as the most frequently reported administrative outcomes. The emphasis on these specific administrative aspects reflects a critical consideration in the implementation of WALANT, highlighting the potential for streamlined processes and enhanced procedural efficiency. This aligns with the broader goal of WALANT to not only provide effective anesthesia but also to contribute to a more efficient and resource-conscious health care environment.

The adoption of WALANT in surgical procedures marks a departure from traditional practices involving tourniquet use for hemostasis. The technical intricacies associated with WALANT have been the subject of scrutiny in seven RCTs. Among these, three studies specifically addressed surgical visibility achieved by pharmacologically induced vasoconstriction. Additionally, two of the seven studies investigated complications as part of their technical outcomes, offering valuable insights into the safety profile of WALANT. Beyond these focal points, the duration of anesthetic effect emerged as another notable outcome explored in these studies, emphasizing the multifaceted nature of the technical considerations associated with the use of WALANT in surgical settings.

## Limitations

Interpretations of this study are limited by the search cutoff date of April 1, 2023, potentially excluding newer relevant studies. The focus on RCTs in inclusion criteria may omit valuable observational studies. The classification of outcomes into five domains and ROB assessment using RoB-2 depend on data reported by authors in the manuscript and may not represent all outcomes evaluated. The absence of established criteria for assessing WALANT RCT outcomes challenges standardization, impacting comparability and reliability across studies.

## Conclusion and Future Strategies

Our systematic review highlighted diverse clinical outcomes in upper limb WALANT RCTs, spanning administrative to functional domains. The Cochrane RoB-2 assessment revealed a low ROB in most studies. Despite this, there is a notable absence of established minimal criteria for evaluating clinical outcomes in WALANT RCTs, underscoring the need for standardized assessment protocols. The need for improved reporting and transparency in RCT studies has also been recognized by the CONSORT group, as a consequence of controversial outcome reporting in clinical trials following the COVID-19 pandemic. An update of the Standard Protocol Items: Recommendations for Interventional Trials and CONSORT guidelines for planning and reporting RCT is underway, which will include methodology and guidelines developed by the enhancing quality and transparency of health research. This comprehensive update is likely to further support researchers and clinicians in the challenging task of conducting randomized trials.^27^

Specific to the field of hand and wrist surgery, a larger global consortium, the International Consortium for Health Outcomes Measurement Hand and Wrist Working Group, has recently provided an in-depth and inclusive report with the aim to standardize patient-centered outcome measures.^28^ Their work provides guidelines for clinical outcome reporting in both surgical and nonsurgical treatment of hand and wrist disorders within different strands based on anatomical area and injury/disease type. Although intended for general clinical outcomes and assessments of patients, these guidelines could well serve as a gold standard in the planning and execution of future RCTs.

## Conflicts of Interest

No benefits in any form have been received or will be received related directly to this article.
